# ESGO/ISUOG/IOTA/ESGE Consensus Statement on preoperative diagnosis of ovarian tumours

**DOI:** 10.52054/FVVO.13.2.016

**Published:** 2021-06-28

**Authors:** D Timmerman, F Planchamp, T Bourne, C Landolfo, A du Bois, L Chiva, D Cibula, N Concin, D Fischerova, W Froyman, G Gallardo, B Lemley, A Loft, L Mereu, P Morice, D Querleu, C Testa, I Vergote, V Vandecaveye, G Scambia, C Fotopoulou

**Affiliations:** Department of Development and Regeneration, KU Leuven, Leuven, Belgium; Department of Obstetrics and Gynaecology, University Hospitals Leuven, Leuven, Belgium; Clinical Research Unit, Institut Bergonie, Bordeaux, France; Department of Metabolism, Digestion and Reproduction, Queen Charlotte’s & Chelsea Hospital, Imperial College, London, UK; Department of Woman, Child and Public Health, Fondazione Policlinico Universitario A. Gemelli IRCCS, Rome, Italy.; Department of Gynaecology and Gynaecological Oncology, Evangelische Kliniken Essen- Mitte, Essen, Germany.; Department of Gynaecology and Obstetrics, University Clinic of Navarra, Madrid, Spain; Department of Obstetrics and Gynaecology, First Faculty of Medicine, Charles University, General University Hospital in Prague, Prague, Czech Republic.; Department of Obstetrics and Gynaecology, Medical University of Innsbruck, Innsbruck, Austria.; Department of Radiology, University Clinic of Navarra, Madrid, Spain.; Patient Representative, President of Kræfti Underlivet (KIU), Denmark; Chair Clinical Trial Project of the European Network of Gynaecological Cancer Advocacy Groups, ENGAGe.; Department of Clinical Physiology, Nuclear Medicine & PET, Rigshospitalet, Copenhagen University Hospital, Copenhagen, Denmark.; Department of Gynaecology and Obstetrics, Gynaecologic Oncology Unit, Santa Chiara Hospital, Trento, Italy.; Department of Gynaecological Surgery, Institut Gustave Roussy, Villejuif, France; Division of Gynaecologic Oncology, Fondazione Policlinico Universitario A Gemelli IRCCS, Rome, Italy; Department of Obstetrics and Gynaecologic Oncology, University Hospital, Strasbourg, France; Institute of Obstetrics and Gynaecology, Università Cattolica del Sacro Cuore, Rome, Italy; Department of Obstetrics and Gynaecology and Gynaecologic Oncology, University Hospital Leuven, Leuven Cancer Institute, Leuven, Belgium.; Department of Radiology, University Hospitals Leuven, Leuven Belgium; Division of Translational MRI, Department of Imaging & Pathology KU Leuven, Leuven, Belgium; Department of Gynaecologic Oncology, Hammersmith Hospital, Imperial College, London, UK

## Abstract

The European Society of Gynaecological Oncology (ESGO), the International Society of Ultrasound in Obstetrics and Gynecology (ISUOG), the International Ovarian Tumour Analysis (IOTA) group and the European Society for Gynaecological Endoscopy (ESGE) jointly developed clinically relevant and evidence-based statements on the preoperative diagnosis of ovarian tumours, including imaging techniques, biomarkers and prediction models.

ESGO/ISUOG/IOTA/ESGE nominated a multidisciplinary international group, including expert practising clinicians and researchers who have demonstrated leadership and expertise in the preoperative diagnosis of ovarian tumours and management of patients with ovarian cancer (19 experts across Europe). A patient representative was also included in the group. To ensure that the statements were evidence-based, the current literature was reviewed and critically appraised.

Preliminary statements were drafted based on the review of the relevant literature. During a conference call, the whole group discussed each preliminary statement and a first round of voting was carried out. Statements were removed when a consensus among group members was not obtained. The voters had the opportunity to provide comments/suggestions with their votes. The statements were then revised accordingly. Another round of voting was carried out according to the same rules to allow the whole group to evaluate the revised version of the statements. The group achieved consensus on 18 statements.

This Consensus Statement presents these ESGO/ISUOG/IOTA/ESGE statements on the preoperative diagnosis of ovarian tumours and the assessment of carcinomatosis, together with a summary of the evidence supporting each statement.

## Introduction

The accurate characterization of newly diagnosed adnexal lesions is of paramount importance to define appropriate treatment pathways. Patients with masses that are suspicious for malignancy should be referred to a gynaecological oncology centre, in order to receive specialist care, as per the definitions of the European Society of Gynaecological Oncology (ESGO) (Querleu et al., 2017) and national and international recommendations and guidelines. For a non-gynaecological primary tumour, patients need to be referred to an appropriate specialist, while patients with benign lesions may be followed up and treated conservatively or may be suitable for less radical surgical treatment, depending on the clinical context (du Bois et al., 2009; Elit et al., 2008; Engelen et al., 2006; Froyman et al., 2019; Vernooij et al., 2007; Woo et al., 2012). Treatment decision-making processes should be based on a combination of the patient’s overall clinical picture, symptoms, preferences, previous medical and surgical history, tumour markers and clinical and radiological findings. A single diagnostic modality alone should not determine the patient’s journey.

The ESGO, the International Society of Ultrasound in Obstetrics and Gynecology (ISUOG), the International Ovarian Tumour Analysis (IOTA) group and the European Society for Gynaecological Endoscopy (ESGE) have, jointly, developed clinically relevant and evidence- based statements on the preoperative diagnosis of ovarian tumours and assessment of disease spread, including imaging techniques, biomarkers and predictive models. Neither screening and follow-up modalities, nor economic analysis of the imaging techniques, biomarkers and prediction models addressed herein, are included within the remit of this Consensus Statement.

## Responsibilities

The present series of statements form a consensus of the authors regarding their currently accepted approaches for the preoperative diagnosis of ovarian tumours and assessment of disease spread, based on the available literature and evidence. Any clinician applying or consulting these statements is expected to use independent medical judgment in the context of individual clinical circumstances to determine all patients’ care and treatment. These statements are presented without any warranty regarding their content, use or application and the authors disclaim any responsibility for their application or use in any way.

## Methods

**Figure 1:**
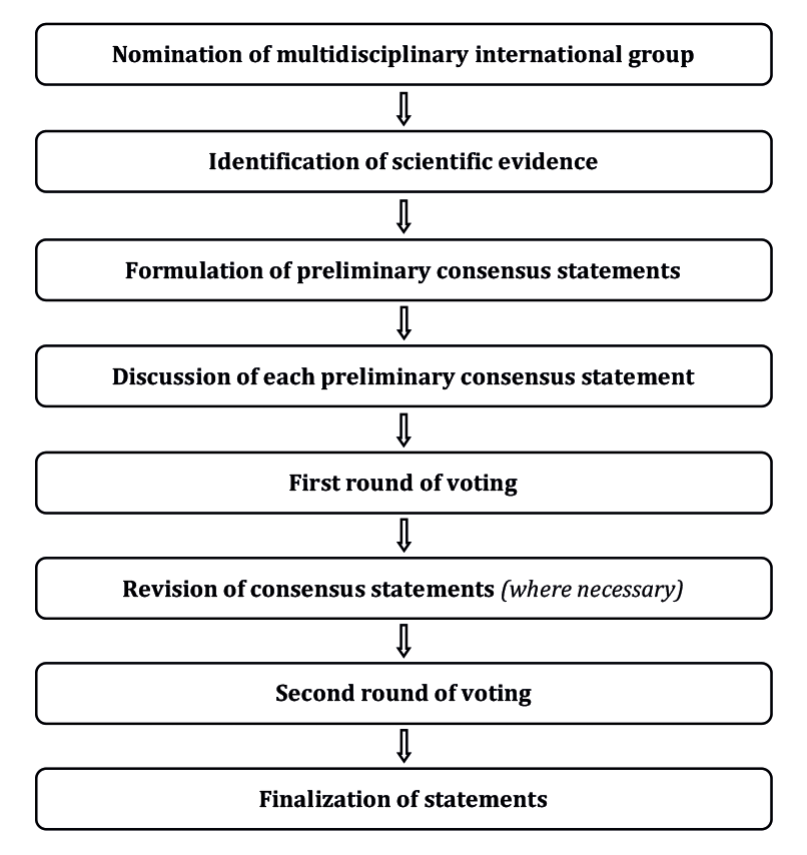
— Eight-step process for development of Consensus Statement on the preoperative diagnosis of ovarian tumours and assessment of disease spread.

This Consensus Statement on the preoperative diagnosis of ovarian tumours and assessment of disease spread was developed using an eight-step process, chaired by Professors Christina Fotopoulou and Dirk Timmerman (Figure 1). Aiming to assemble a multidisciplinary international group, ESGO/ISUOG/IOTA/ESGE nominated 19 practising clinicians and researchers who have demonstrated leadership and expertise in the preoperative diagnosis of ovarian tumours and clinical management of ovarian cancer patients through research, administrative responsibilities, and/or committee membership (including eight members of ESGO, five members of ISUOG, four members of IOTA and two members of ESGE).

These experts included seven gynaecologists with special interest in ultrasonography, two radiologists and 10 gynaecological oncologists. They did not represent the societies from which they were selected, and were asked to base their decisions on their own experience and expertise. Also included in the group was a patient representative, who is Chair of the Clinical Trial Project of the European Network of Gynaecological Cancer Advocacy Groups, ENGAGe. An initial conference call, including the whole group, was held to facilitate introductions, as well as to review the purpose and scope of this Consensus Statement.

To ensure that the statements were evidence- based, the current literature was reviewed and critically appraised. Thus, a systematic literature review of relevant studies published between 1 May 2015 and 1 May 2020 was carried out using the MEDLINE database (Appendix 1). The literature search was limited to publications in the English language. Priority was given to high-quality systematic reviews, meta-analyses and validating cohort studies, although studies with lower levels of evidence were also evaluated. The search strategy excluded editorials, letters and case reports. The reference list of each identified article was reviewed for other potentially relevant articles. Final results of the literature search were distributed to the whole group, including electronic full-text versions of each article. F. Planchamp provided the methodology and medical writing support for the entire process, and did not participate in voting for statements.

The chairs were responsible for drafting preliminary statements based on the review of the relevant literature. These were then sent to the multidisciplinary international group prior to a second conference call. During this conference call, the whole group discussed each preliminary statement and a first round of binary voting (agree/disagree) was carried out for each potential statement. All 20 participants took part in each vote, but they were permitted to abstain from voting if they felt they had insufficient expertise to agree/disagree with the statement or if they had a conflict of interest that could be considered to influence their vote. Statements were removed when a consensus among group members was not obtained. The voters had the opportunity to provide comments/suggestions with their votes. The chairs then discussed the results of this first round of voting and revised the statements if necessary. The voting results and the revised version of the statements were again sent to the whole group and another round of binary voting was organized, according to the same rules, to allow the whole group to evaluate the revised version of the statements. The statements were finalized based on the results of this second round of voting. The group achieved consensus on 18 statements. In this Consensus Statement, we present a summary of the supporting evidence, the finalised series of statements, and their levels of evidence and grades.

## Results

### General remarks

Even though the test performance of any biochemical or radiological diagnostic test appears to increase after excluding borderline ovarian tumours and non-gynaecological primary tumours, such as of the gastrointestinal tract or breast, we included in our literature assessment studies addressing all types of adnexal tumour, as this is a better reflection of clinical reality.

### Ultrasonography

#### 

A transvaginal ultrasound examination is often regarded in clinical practice as the standard first- line imaging investigation for the assessment of adnexal pathology (Kaijser et al., 2014; Meys et al., 2016; Timmerman, 2004; Valentin et al., 2001). The diagnostic accuracy of ultrasonography in differentiating between benign and malignant adnexal masses has been shown to relate to the expertise of the operator (Timmerman et al., 1999; Valentin, 1999; Yazbek et al., 2008). The European Federation of Societies for Ultrasound in Medicine and Biology has published minimum training requirements for gynaecological ultrasound practice in Europe, including standards for theoretical knowledge and practical skills (Education and Practical Standards Committee, European Federation of Societies for Ultrasound in Medicine and Biology, 2006). These identify three levels of training and expertise. Thus, Level-III (expert) can be attributed to a practitioner who is likely to spend the majority of their time undertaking gynaecological ultrasound and/or teaching, research and development in the field. A Level-II practitioner should have undertaken at least 2000 gynaecological ultrasound examinations. The training required to attain this level of practice would usually be gained during a period of expert ultrasound training, which may be within, or after completion of, a specialist training program. To maintain competence at Level-II, practitioners should perform at least 500 examinations each year. A Level-I practitioner should have performed a minimum of 300 examinations under the supervision of a Level-II practitioner or an experienced Level-I practitioner with at least 2 years’ regular practical experience. To maintain Level-I status, the practitioner should perform at least 300 examinations each year. A prospective randomized controlled trial to assess the effect of the quality of gynaecological ultrasonography on the management of patients with suspected ovarian cancer has demonstrated that women with a Level- III (expert) ultrasound examination undergo significantly fewer unnecessary major procedures and have a shorter inpatient hospital stay compared with those having a Level-II (routine) examination by a sonographer (Yazbek et al., 2008).

Subjective assessment by expert ultrasound examiners has excellent performance to distinguish between benign and malignant ovarian tumours (Meys et al., 2016; Timmerman, 2004; Timmerman et al., 1999; Valentin, 1999; Valentin et al., 2001; Yazbek et al., 2008). In many cases, expert examiners should be able to narrow the diagnosis down further, to a specific histological subtype. The typical pathognomonic ultrasound features of some key histological types have been published in the series, ‘Imaging in gynecological disease’, in Ultrasound in Obstetrics and Gynecology. The most common and typical findings for each pathology are summarized in Table I.

**Table I:** Clinical and ultrasound features typical of different histological subtypes of adnexal tumour.

Category/Type	Age (years)	Laterality	Appearance	Typical features	Colour score	Image	Ref
Endometriosis-related tumours							
Endometrioma	Median, 34	Uni/bi	Uni- or multilocular (1–4 locules)	Ground-glass content; papillations in 10%, but most often without internal blood flow; premenopausal patient; raised CA 125 (median, 44U/mL)	1/2/(3)	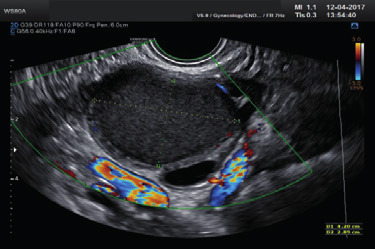	171
Benign tumours							
Sex cord-stromal tumour							
Fibroma/fibrothecoma (65%)	Median, 50; 65% postmenopausal	Uni	Regular round, oval or slightly lobulated solid tumours; sometimes multilocular-solid (15–20%)	Fan-shaped shadowing; often, raised CA 125 (34%) and/or ascites	(1)/2/3	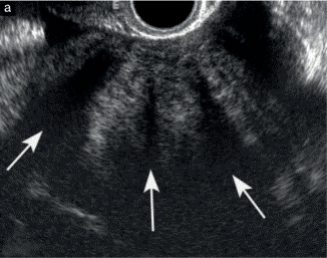	172
Sertoli-cell tumour (most benign)	≤ 30 (75%)	Uni	Solid (median diameter, 90 mm)	Hormonally inactive or oestrogen- producing (abnormal bleeding)	3/4	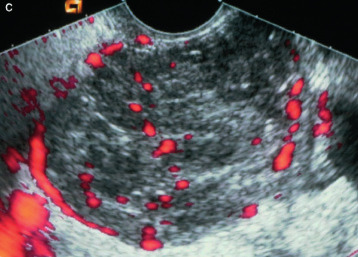	173
Leydig-cell tumour (almost all benign)	Median, 58	Uni	Solid (median diameter, 24 mm)	Endocrine symptoms (75% virilisation); testosterone/ androstenedione	3/4	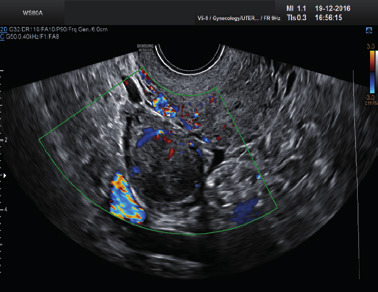	173
Germ-cell tumour							
Mature cystic teratoma (dermoid)	Median, 33	Uni (88%)	Uni- (58%) or multilocular (or uni-/ multilocular solid)	Mixed echogenicity/ white ball and stripes/shadowing; CA 19-9 elevated in 30%	1/2/(3)	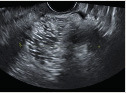	†
Struma ovarii (entirely or predominantly thyroid tissue); 3% of all ovarian teratomas	Median, 40	Uni/bi	Multilocular/multilocular solid; rarely, papillations; fluid anechoic or low-level	‘Struma pearl’: smooth; roundish solid area; thyrotoxicosis may occur	1/2/3	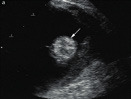	174
Epithelial							
Serous cystadenoma	40–60	Uni (80–90%)	Uni- or multilocular (2–10 locules)	Anechoic cystic fluid; often, papillations without internal blood flow	1/2	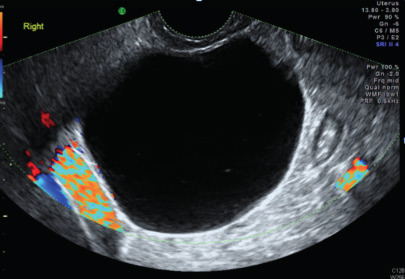	‡
Serous cystadenofibroma	40–60	Uni (84%)	Multilocular-solid (37%), unilocular- solid (30%), multilocular (19%) or unilocular (13%); median diameter, 50– 80 mm	One (52%), two (17%) or three (13%) papillations; absent colour Doppler signals (80%) and shadows behind papillations (40%)	1/2	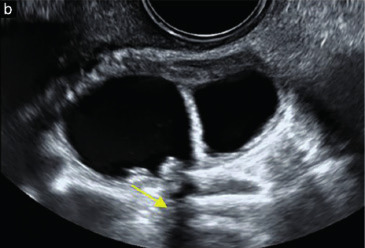	175
Mucinous cystadenoma	Median, 50	Uni (95%)	Multilocular (65%) > 10 locules; sometimes unilocular (18%) or multilocular-solid (16%); median diameter, 112 mm	Sometimes ‘honeycomb nodule’	1/2/(3)	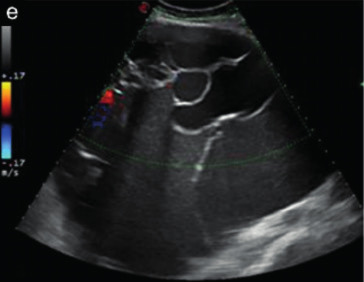	176
Brenner tumour (99% benign)	30–70	Uni	Small solid tumours, 20–80 mm; often extensive calcifications; sometimes multilocular-solid	Small cysts often seen in solid tumours; shadowing; CA 125 raised in 10%	1/2/(3)	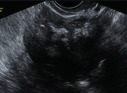	177
Tumour-like lesions							
Infection							
Abscess	16–50	Uni/bi	Uni-/multilocular	Cogwheel appearance; mixed echogenicity; acute pain; raised CA 125	3/4	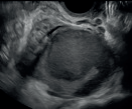	178
Malignant tumours							
Epithelial							
Borderline serous	Median, 42; 30% < 40	Uni (73%)/ bi (27%)	Unilocular-solid (55%) or multilocular-solid (30%); cystic fluid anechoic (47%) or low-level	> 3 irregular papillations (81%) with internal blood flow and anechoic spaces; no shadowing	2/3	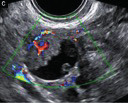	179,180,181
Borderline mucinous (intestinal type) (30–50%)	Median, 50	Uni	Multilocular (80%) or unilocular (15%); very large tumour ( median diameter, 195 mm)	Multiple small loculi, often ‘honeycomb nodule’; no papillations; cystic fluid low-level	2/3	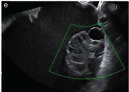	176,179
Borderline mucinous (endocervical type)	30–40	Uni	Unilocular-solid; sometimes multilocular-solid; median diameter, 37 mm	Papillations (60%); cystic fluid low-level or ground-glass	2/3	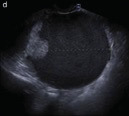	176,179
Borderline seromucinous (new category)	Median 42	Uni	Contain endometrioid-, indifferent- and squamous-type epithelium	Frequently associated with endometriosis	—	—	176,179
Low-grade serous carcinoma	Median, 53	Bi (60%)	Multilocular-solid (55%) or solid (32%)	Small calcifications in solid tissue; papillations (32%)	2/3/4	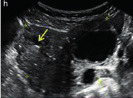	180
High-grade serous carcinoma	55–65	Bi (50%)	Solid (64%) or multilocular-solid (33%)	Areas of necrosis in solid tissue; rarely, papillations (7%)	2/3/4	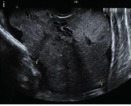	180
Mucinous carcinoma (3%)	Median, 53	Uni (80%)	Multilocular-solid (55%), multilocular or solid	Very large tumour (median diameter, 197 mm); cystic fluid low-level	2/3/(4)	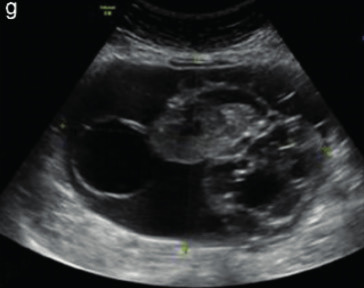	176
Endometrioid carcinoma (10–15%)	Median, 55	Uni (79%); coexist with endometrial carcinoma (20%)	Multilocular- solid (48%) with low-level (53%) or ground-glass (16%) cystic fluid, or solid (34%); median diameter, 102 mm	Cockade-like appearance; papillations in 29%; 20% develop from endometriosis	(2)/3/4	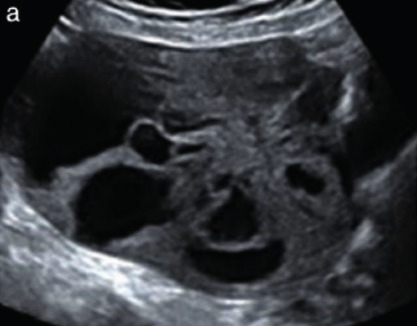	183
Clear-cell carcinoma (5-25%)	Median, 55	Uni (85%)	Multilocular- solid (41%), or unilocular-solid (35%) with low- level (44%) or ground-glass (22%) cystic fluid, or solid (24%); median diameter, 117 mm	Solid nodules; papillations in 38%; 20–30% develop from endometriosis	(2)/3/4	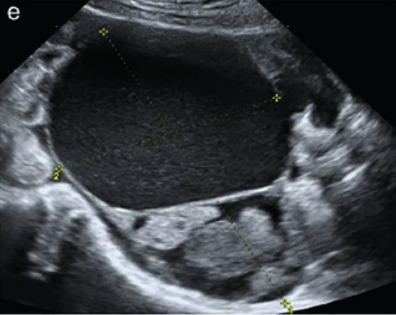	184
Carcinosarcoma	Median, 66 (range, 33–91)	Bi (50%)	Solid (72.5%); multilocular-solid (24.5%); median diameter, 100 mm	Most tumours solid with irregular margins and cystic areas	3/4	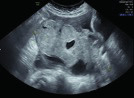	§
Sex cord-stromal tumour							
Granulosa-cell tumour (70%)	50% premenopause; 3–10% prepubertal (juvenile type)	Uni	Large multilocular- solid/solid (median diameter, 100 mm); heterogeneous solid tissue with areas of necrosis and haemorrhage; echogenicity of fluid mixed or low-level; rarely, papillations	‘Swiss cheese’ pattern; hyperoestrogenic (abnormal bleeding, thick endometrium); CA 125 normal; oestradiol elevated in postmenopause	3/4	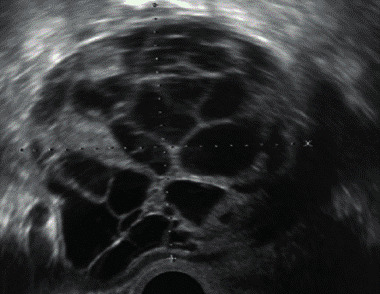	185
Sertoli-Leydig-cell tumour	≤ 30 (75%)	Uni (100%)	Large multilocular- solid or solid (median diameter, 50-150 mm)	Endocrine symptoms (one third virilisation); testosterone/ androstenedione	3/4	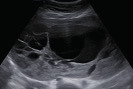	173
Germ-cell tumour							
Dysgerminoma	Median, 20 (range, 16–31)	Uni	Highly vascularized, purely solid tumours with heterogeneous internal echogenicity divided into several lobules; smooth and sometimes lobulated contour; well-defined relative to surrounding organs	Internal lobular appearance; raised LDH, sometimes AFP	3/4	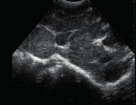	186
Yolk-sac tumour	20–30	Uni	Large and irregular multilocular-solid/ solid (100–200 mm)	Fine-textured slightly hyperechoic solid tissue; raised AFP	3/4	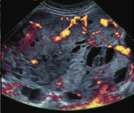	187,188
Immature teratoma	15–30	Uni	Large, predominantly solid	Very inhomogeneous solid tissue with hyper-refl ective areas; raised AFP	2/3/4	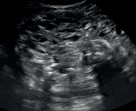	¶
Endodermal-sinus tumour*	—	—	—	Always raised AFP	—	—	—
Choriocarcinoma	Median, 36	Uni	Large, solid (inhomogeneous echogenicity) with small and irregular cystic spaces	Raised hCG	(3)/4	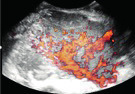	189
Embryonal carcinoma	14–20	Uni	Large, solid (inhomogeneous echogenicity) with small and irregular cystic spaces	Raised hCG and AFP	(3)/4	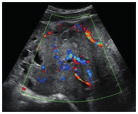	189
Malignant mixed germ-cell tumour	Median, 18	Uni	Large, solid (inhomogeneous echogenicity) with small and irregular cystic spaces	Raised hCG/LDH/AFP	(3)/4	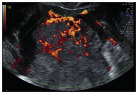	189
Secondary metastatic							
Breast, stomach, lymphoma or uterus	Median, 56	Bi (50–75%)/uni	Solid (median diameter, 70 mm)	‘Lead-vessel’ sign; CA 125 moderately raised in 75%; CA 15-3 raised (breast)	(3)/4	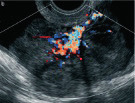	190
Colon, rectum, appendix or biliary tract	Median, 56; appendix younger (25–50)	Bi (50–75%)/uni	Multilocular/ multilocular-solid (median diameter, 120 mm); many locules; irregular; papillations	CA 125 moderately raised in 75%; CEA raised (colon, rectum)/CA19-9 raised (biliary tract)	(2)/3/(4)	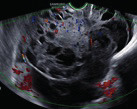	190
Tumour of Fallopian tube: epithelial							
Tubal cancer	55–60	Uni (90%)	Completely solid or with large solid component(s) and anechoic cystic fluid; average, 50 mm	Well-vascularized ovoid or sausage- shaped structure; normal ovarian tissue adjacent in 50%	3/4	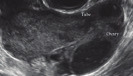	191

All example images in this table are reproduced from the cited references in Ultrasound in Obstetrics and Gynaecology.

*Yolk-sac tumour. †Heremans et al. (pers. comm.). ‡Virgilio et al. (pers. comm.). §Ciccarone et al. (pers. comm.). ¶ Landolfo et al. (pers. comm.).

AFP, alpha-fetoprotein; Bi, bilateral; hCG, human chorionic gonadotrophin; LDH, lactate dehydrogenase; Ref, reference; Uni, unilateral.

#### Risk of malignancy index (RMI) and risk of ovarian malignancy algorithm (ROMA)

Several attempts have been made to develop more objective ultrasound-based approaches for discriminating between benign and malignant adnexal tumours. These include the risk of malignancy index (RMI), a scoring system based on menopausal status, a transvaginal ultrasound score and serum cancer antigen 125 (CA 125) level (Jacobs et al., 1990). Many studies have demonstrated the diagnostic performance of the RMI in classifying adnexal masses (Akturk et al., 2011; Al-Musalhi et al., 2015; Al Musalhi et al., 2016; Anton et al., 2012; Bouzari et al., 2011; Chacon et al., 2019; Chopra et al., 2015; Dochez et al., 2019; Hada et al., 2020; Javdekar & Maitra, 2015; Khoiwal et al., 2019; Meys et al., 2016; Westwood et al., 2018; Zhang et al., 2019). Three variants of the RMI (RMI-II, RMI-III, RMI-IV) have been developed, but these offer no significant additional diagnostic advantage compared with the original version (RMI-I) (Akturk et al., 2011; Hada et al., 2020; Meys et al., 2016; Zhang et al., 2019). Moore et al. (2008) developed an algorithm, the risk of ovarian malignancy algorithm (ROMA), based on both CA 125 and human epididymis protein 4 (HE4). Westwood et al. (2018) pooled data comparing the ROMA with the RMI-I to guide referral decisions for women with suspected ovarian cancer and found similar performance if women with borderline tumours and non-epithelial cancers were excluded from the analyses. More recently, another meta-analysis showed a higher specificity of the RMI-I than the ROMA in premenopausal women but a similar performance for detecting ovarian cancer in postmenopausal women presenting with an adnexal mass (Chacon et al., 2019). Limitations of the RMI are the absence of an estimated risk of malignancy, and its considerable dependence on serum CA 125, the latter resulting in a relatively low sensitivity for early-stage invasive and borderline disease, especially in premenopausal women (Kaijser et al., 2013; Timmerman et al., 2007) (see Tumour Markers).

#### IOTA methods

To homogenize and standardize the quality, description and evaluation of ultrasonography across different centres, and thereby increase diagnostic accuracy, the IOTA group first published a consensus paper on terms and definitions to describe adnexal lesions in 20003 (Timmerman et al., 2016). Using this standardized methodology, the IOTA group has developed different prediction models based on logistic regression analysis (Timmerman et al., 2005; Timmerman et al., 2016; Van Calster et al., 2014). In a large-scale external validation study, Van Holsbeke et al. (2012) showed that the IOTA logistic regression models 1 (LR1, with 12 variables) and 2 (LR2, with six variables) outperformed 12 other models, including the RMI. The LR2 model was easier to use than the LR1 model. Demonstrating the standardization and reproducibility of the IOTA models, Sayasneh et al. (2013) showed that even less-experienced sonographers are able to differentiate accurately between benign and malignant ovarian masses using the IOTA LR1 model. The IOTA group also developed ‘Simple Rules’ that may be applied to a mass based on the presence or absence of five benign and five malignant ultrasound features. These rules can be applied to about 80% of adnexal masses, with the rest being classed as inconclusive. They have now been broadly accepted and are widely used in clinical practice (Alcazar et al., 2013; Hartman et al., 2012; Knafel et al., 2016; Nunes et al., 2014; Ruiz de Gauna et al., 2015; Sayasneh et al., 2013; Tantipalakorn et al., 2014; Timmerman et al., 2010; Timmerman et al., 2008). More recently, a logistic regression model based on the ultrasound features of the original Simple Rules was developed, i.e. the Simple Rules risk model. This model is able to provide an individual estimated risk of malignancy for any type of lesion (Timmerman et al., 2016). A summary of the main models and scoring systems for the preoperative diagnosis of ovarian tumours is presented in Table II.

**Table II:** ­– Summary of main models and scoring systems for preoperative diagnosis of ovarian tumors.

Model or system: type	Predictor variables	Remarks
Simple descriptors : classification as benign or malignant	Benign descriptor (BD) 1: Unilocular tumour with ground-glass echogenicity in a premenopausal woman; BD2: Unilocular tumour with mixed echogenicity and acoustic shadows in a premenopausal woman; BD3 Unilocular anechoic tumour with regular walls and maximum diameter of lesion < 10 cm; BD4 Remaining unilocular tumour with regular walls; Malignant descriptor (MD) 1: Tumour with ascites and at least moderate colour Doppler blood flow in a postmenopausal woman; MD2 Age > 50 years and CA 125 > 100 U/mL	No risk estimates Based on clinical, ultrasound and CA 125 information Possible to calculate result without computer
RMI : score	CA 125, menopausal status, ultrasound score based on five binary ultrasound variables (multilocular cyst, solid areas, bilateral lesions, ascites, evidence of metastases on abdominal ultrasound)	No risk estimates Based on clinical, ultrasound and CA 125 information Possible to calculate result without computer Online calculators available
Simple Rules : classification as benign, inconclusive or malignant	Classification based on 10 binary features, i.e. five benign and five malignant features: Benign features: unilocular cyst, smooth multilocular cyst with largest diameter < 100 mm, presence of solid areas with largest diameter < 7 mm, acoustic shadows, no vascularization on colour Doppler Malignant features: irregular solid tumour, irregular multilocular solid tumour with largest diameter ≥ 100mm, presence of ascites, ≥ 4 papillary projections, very strong vascularization on colour Doppler	No risk estimates Classification into only three groups Based on dichotomized ultrasound features Easy to use without computer Available as smartphone app
LR2: risk model based on logistic regression	Age (years), presence of acoustic shadows, presence of ascites, presence of papillary projections with blood flow, maximum diameter of largest solid component, irregular internal cyst walls	Risk estimates Based on clinical and ultrasound information Requires computer Available as smartphone app
Simple Rules risk: risk model based on logistic regression	The 10 binary features used in the Simple Rules, type of centre (oncology centre vs other)	Risk estimates Based on dichotomized ultrasound features Developed to add risk estimates for Simple Rules Available as online calculator; available in ultrasound machines from some manufacturers
ADNEX without CA 125 : risk model based on multinomial logistic regression	Age (years), maximum diameter of lesion (mm), maximum diameter of largest solid component (mm), number of papillary projections (ordinal), presence of acoustic shadows, presence of ascites, presence of more than 10 cyst locules, type of centre (oncology centre vs other)	Risk estimates Also estimates risk of four subtypes of malignancy Based on clinical and ultrasound information Subjective predictors are avoided *a priori* (e.g. colour score or irregular cyst walls) Requires computer Available as smartphone app and as online calculator; available in ultrasound machines from some manufacturers
ADNEX with CA 125: risk model based on multinomial logistic regression	Same variables as for ADNEX without CA 125, and additionally serum CA 125 (IU/L)	Risk estimates Also estimates risk of four subtypes of malignancy Based on clinical, ultrasound and CA 125 information Subjective predictors are avoided *a priori* (e.g. colour score or irregular cyst walls) Requires computer Available as smartphone app and as online calculator; available in ultrasound machines from some manufacturers

As many ovarian masses can be recognized relatively easily, the IOTA group also proposed four ‘Simple Descriptors’ of the features typical of common benign lesions and two suggestive of malignancy, which can give an ‘instant diagnosis’ and reflect the pattern recognition that is a key part of ultrasonography. These are applicable to about 43% of adnexal masses (Ameye et al., 2012). A three-step strategy, consisting of the sequential use of Simple Descriptors, Simple Rules and subjective assessment by an expert, had high accuracy for discriminating between benign and malignant adnexal lesions (Ameye et al., 2012). A systematic review and meta-analysis reported better performance of the IOTA Simple Rules and the IOTA LR2 model compared with all other scoring systems, including the RMI (Kaijser et al., 2014). Besides confirming these findings, another meta-analysis highlighted that a two-step approach, with the IOTA Simple Rules as the first step and subjective assessment by an expert for inconclusive tumours as the second step, matched the test performance of expert ultrasound examiners (Meys et al., 2016). The IOTA Simple Rules have been integrated into several national clinical guidelines for the evaluation and management of adnexal masses (American College of Obstetricians and Gynecologists’ Committee on Practice Bulletins, 2016) and they were considered the main diagnostic strategy (Glanc et al., 2017) as part of a first international consensus report for the assessment of adnexal masses.

A randomized controlled trial assessing surgical intervention rates and the oncologic safety of decision-making processes using on an RMI-based protocol developed by the British Royal College of Obstetricians and Gynaecologists (RCOG) vs triage using the IOTA Simple Rules (Nunes et al., 2017) showed that the IOTA protocol resulted in lower surgical intervention rates compared with the RMI-based RCOG protocol. The IOTA Simple Rules did not result in more cases in which a diagnosis of cancer was delayed. It was found that the addition of biomarkers such as serum CA 125 and HE4 when using the IOTA Simple Rules, with or without subjective assessment by an expert sonographer, offered no additional diagnostic advantage for the characterization of ovarian masses, but was more costly than a three- step strategy based on the sequential use of the IOTA Simple Descriptors, Simple Rules and expert evaluation (Alcazar et al., 2016; Piovano et al., 2017).

The IOTA group have also developed the Assessment of Different NEoplasias in the adneXa (ADNEX) model. This multiclass prediction model is the first risk model to differentiate between benign and malignant tumours, whilst also offering subclassification of any malignancy into borderline tumours, Stage-I and Stage-II– IV primary cancers and secondary metastatic tumours. The IOTA ADNEX model was developed and validated using parameters collected by experienced ultrasound examiners (Van Calster et al., 2014). Several external validation studies have shown good to excellent performance of the ADNEX model in discriminating different types of ovarian tumour, with a higher clinical value than the RMI (Araujo et al., 2017; Meys et al., 2017; Sayasneh et al., 2016; Szubert et al., 2016; Van Calster, 2017; Van Calster et al., 2016; Wynants et al., 2017). A study aiming to validate the ADNEX model when applied by Level-II examiners has confirmed that it can be used successfully by less-experienced examiners (Viora et al., 2020). A large multicentre cohort study of 4905 masses in 17 centres, comparing six different prediction models (RMI, LR2, Simple Rules, Simple Rules risk model and ADNEX model with or without CA 125), demonstrated the IOTA ADNEX model and the IOTA Simple Rules risk model to be the best models for the characterization of ovarian masses in patients who present with an adnexal lesion (Van Calster et al., 2020).

#### GI-RADS

The Gynaecologic Imaging Reporting and Data System (GI-RADS) was first introduced by Amor et al. in 2009 and was validated prospectively by the same team in a multicentre study 2 years later (Amor et al., 2011; Amor et al., 2009). This reporting system quantifies the risk of malignancy into five categories: GI-RADS 1, definitively benign (estimated probability of malignancy (EPM) = 0%); GI-RADS 2, very probably benign (EPM < 1%); GI-RADS 3, probably benign (EPM = 1–4%); GI-RADS 4, probably malignant (EPM = 5–20%); and GI-RADS 5, very probably malignant (EPM > 20%). More recently, several studies have demonstrated the value of the GI- RADS system for the assessment of malignant adnexal masses in women who are candidates for surgical intervention. Furthermore, the addition of GI-RADS to CA 125 improves the identification of adnexal masses at high risk of malignancy compared with using CA 125 alone (Basha et al., 2019; Behnamfar et al., 2019; Koneczny et al., 2017; Migda et al., 2018; Zhang et al., 2017; Zheng et al., 2019).

#### O-RADS

The Ovarian-Adnexal Reporting and Data System (O-RADS) lexicon for ultrasound was published in 2018, providing a standardized glossary that includes all appropriate descriptors and definitions of the characteristic ultrasound appearance of normal ovaries and various adnexal lesions (Andreotti et al. 2018; 2019). The O-RADS ultrasound working group developed an adnexal- mass triage system based either on the O-RADS descriptors or on the risk of malignancy assigned to the mass using the IOTA ADNEX model to classify ovarian tumours into different risk categories (Andreotti et al., 2020). However, to date, neither the triage system nor the O-RADS descriptors have been externally validated. Basha et al. (2020) determined the malignancy rates, validity and reliability of the O-RADS approach when applied to a database of 647 adnexal masses collected before the development of the O-RADS system. In this retrospective study, the O-RADS system had significantly higher sensitivity than did the GI-RADS system and the IOTA Simple Rules, with a non-significant slightly lower specificity compared with both GI-RADS and IOTA Simple Rules, and with similar reliability.

**Statements on ultrasonography (Statements 1–6)**

1.
  Subjective assessment by expert (Level-III) ultrasound examiners has the best performance to distinguish between benign and malignant ovarian tumours.
  - Level of evidence: 1a
  - Grade of statement: A
  - Consensus: yes, 95% (n = 19); no, 0% (n = 0); abstain, 5% (n = 1)
2.
  If an expert ultrasound examiner is not available, the use of ultrasound-based diagnostic models can assist clinicians to distinguish between benign and malignant ovarian tumours.
  - Level of evidence: 2a
  - Grade of statement: B
  - Consensus: yes, 90% (n = 18); no, 0% (n = 0); abstain, 10% (n = 2)
3.
  Ultrasound-based diagnostic models (IOTA Simple Rules risk model or IOTA ADNEX model) are preferable to CA 125 level, HE4 level or ROMA as they are superior in distinguishing between benign and malignant ovarian tumours.
  - Level of evidence: 2b
  - Grade of statement: B
  - Consensus: yes, 95% (n = 19); no, 0% (n = 0); abstain, 5% (n = 1)
4.
  The IOTA ADNEX model and the IOTA Simple Rules risk model are recommended as they outperform existing morphological scoring systems, including the RMI.
  - Level of evidence: 1b
  - Grade of statement: A
  - Consensus: yes, 95% (n = 19); no, 0% (n = 0); abstain, 5% (n = 1)
5.
  The IOTA ADNEX model is a multiclass model and is helpful to differentiate between benign tumours, borderline tumours, early- or advanced- stage ovarian cancer and secondary metastatic tumours.
  - Level of evidence: 3b
  - Grade of statement: C
  - Consensus: yes, 85% (n = 17); no, 0% (n = 0); abstain, 15% (n = 3)
6.
  The threshold risk of there being a secondary metastatic tumour (as predicted by the IOTA ADNEX model), above which additional investigations to detect the primary organ of origin should be triggered, is 10%.
  - Level of evidence: 5
  - Grade of statement: D
  - Consensus: 5% threshold, 10% (n = 2); 10% threshold, 75% (n = 15); 15% threshold, 0% (n = 0); 20% threshold, 0% (n = 0); abstain, 15% (n = 3)

Levels of evidence and grades are described in Table III.

**Table III:** ­Levels of evidence and grades of statement used in this Consensus Statement..

Levels of evidence		
1a	Systematic review (with homogeneity) of Level-1 diagnostic studies; or clinical decision rule with Level-1b studies from different clinical centres	
1b	Validating cohort study with good reference standards; or clinical decision rule tested within one clinical centre	
1c	Absolute SpPins and SnNouts*	
2a	Systematic review (with homogeneity) of Level > 2 diagnostic studies	
2b	Exploratory cohort study with good reference standards; or clinical decision rule after derivation, or validated only on split-sample or databases	
3a	Systematic review (with homogeneity) of studies Level ≥ 3b	
3b	Non-consecutive study; or without consistently applied reference standards	
4	Case–control study, poor or non-independent reference standard	
5	Expert opinion without explicit critical appraisal, or based on physiology, bench research or ‘first principles’	
Grades of statement		
Code	Quality of evidence	Definition
A	High	Further research is very unlikely to change our confidence in the estimate of effect. ● Several high-quality studies with consistent results ● In special cases: one large, high-quality multicentre trial
B	Moderate	Further research is likely to have an important impact on our confidence in the estimate of effect and may change the estimate. ● One high-quality study ● Several studies with some limitations
C	Low	Further research is very likely to have an important impact on our confidence in the estimate of effect and is likely to change the estimate. ● One or more studies with severe limitations
D	Very low	Any estimate of effect is very uncertain. ● Expert opinion ● No direct research evidence ● One or more studies with very severe limitations

Note: A minus sign ‘–‘ may be added to denote evidence that fails to provide a conclusive answer because it is either (a) a single result with a wide confidence interval; or (b) a systematic review with considerable heterogeneity. Such evidence is inconclusive, and therefore can only generate Grade D recommendations. *’Absolute SpPin’ is a diagnostic finding whose specificity is so high that a positive result rules in the diagnosis; ‘Absolute SnNout’ is a diagnostic finding whose sensitivity is so high that a negative result rules out the diagnosis.

#### 

According to a systematic quantitative review assessing the accuracy of CA 125 level in the diagnosis of benign, borderline and malignant ovarian tumours, CA 125 is the best available single-protein biomarker identified to date (Medeiros et al., 2009). Although it lacks sensitivity and specificity for early stages of the disease and has a relatively low specificity overall, it can help direct treatment options in patients with suspicious ovarian masses. Pooled analyses have highlighted that a high body mass index and ethnicity might influence CA 125 levels, representing an additional diagnostic challenge (Babic et al., 2017). Other factors that influence CA 125 levels are the age of the patient, pregnancy, inflammatory processes and the presence of fibroids or endometriosis (Babic et al., 2017; Cramer et al., 2010; Johnson et al., 2008; Pauler et al., 2001).

Multiple studies, including meta-analyses, have highlighted the role of HE4 as a potential complement to CA 125, especially in differentiating benign endometriotic and inflammatory lesions in younger women (Al Musalhi et al., 2016; Cao et al., 2018; Huang et al., 2018; Jacob et al., 2011; Jia et al., 2017; Kim et al., 2019; Kotowicz et al., 2015; Li et al., 2012; Lin et al., 2013; Lycke et al., 2018; Melo et al., 2018; Richards et al., 2015; Romagnolo et al., 2016; Sandri et al., 2013; Shin et al., 2020; Stiekema et al., 2014; Terlikowska et al., 2016; Van Gorp et al., 2012; Wang et al., 2014; Xu et al., 2016; Yanaranop et al., 2017; Yanaranop et al., 2018; Yu et al., 2012; Zhang et al., 2015). Additional tumour markers (as in the ROMA test) have failed to improve significantly the discrimination between benign and malignant masses compared with CA 125 alone (Chen et al., 2015; Choi et al., 2020; Cui et al., 2019; Huy et al., 2018; Kaijser, Van Gorp, et al., 2014; Kim et al., 2019; Kotowicz et al., 2015; Lycke et al., 2018; Melo et al., 2018; Piovano et al., 2017; Romagnolo et al., 2016; Sandri et al., 2013; Shen et al., 2017; Shin et al., 2020; Terlikowska et al., 2016; Xu et al., 2016; Yanaranop et al., 2018; Zhang et al., 2015). The combination of a more extended tumour marker profile, including the addition of carcinoembryonic antigen (CEA) and/ or carbohydrate antigen (CA 19-9) to CA 125, is useful mainly for differentiating between metastatic tumours from the gastrointestinal tract or pancreas and primary ovarian malignancy (Bozkurt et al., 2013; Kelly et al., 2010; Sagi-Dain et al., 2015a, 2015b).

**Statements on tumour markers (Statements 7–12)**

-
  CA 125 is the best single-protein biomarker for the preoperative characterization of ovarian tumours. However, it is not useful as a screening test for ovarian cancer.
  - Level of evidence: 2b
  - Grade of statement: B
  - Consensus: yes, 95% (n = 19); no, 0% (n = 0); abstain, 5% (n = 1)
-
  Neither HE4 nor ROMA improves the discrimination between benign and malignant masses compared with CA 125 alone.
  - Level of evidence: 2b
  - Grade of statement: B
  - Consensus: yes, 70% (n = 14); no, 0% (n = 0); abstain, 30% (n = 6)
-
  CA 125 does not increase the performance of ultrasound-based risk models to distinguish between benign and malignant tumours.
  - Level of evidence: 2b
  - Grade of statement: B
  - Consensus: yes, 60% (n = 12); no, 10% (n = 2); abstain, 30% (n = 6)
-
  CA 125 is helpful as a biomarker in cases of suspected malignancy and it helps to distinguish between subtypes of malignant tumours, such as borderline and early- and advanced-stage primary ovarian cancers and secondary metastatic tumours.
  - Level of evidence: 2b
  - Grade of statement: B
  - Consensus: yes, 90% (n = 18); no, 5% (n = 1); abstain, 5% (n = 1)
-
  CEA may be useful in specific cases to differentiate between primary ovarian cancer and secondary (ovarian) tumours.
  - Level of evidence: 3b
  - Grade of statement: C
  - Consensus: yes, 90% (n = 18); no, 0% (n = 0); abstain, 10% (n = 2)
-
  CA 19-9 can help to differentiate secondary metastatic tumours in the ovary.
  - Level of evidence: 3b
  - Grade of statement: C
  - Consensus: yes, 75% (n = 15); no, 5% (n = 1); abstain, 20% (n = 4)

Levels of evidence and grades are described in Table III.

### Magnetic resonance imaging / computed tomography / positron emission tomography- computed tomography

#### Magnetic resonance imaging

Several reports have found that magnetic resonance imaging (MRI), alone or in combination with computed tomography (CT), predicts accurately the presence of peritoneal carcinomatosis in patients undergoing preoperative evaluation for cytoreductive surgery, particularly when the assessment is carried out by an experienced radiologist (Dohan et al., 2017; Gadelhak et al., 2019; Low et al., 2015; Torkzad et al., 2015). Recently, a prospective study reported higher specificity of the IOTA LR2 model compared with subjective interpretation of MRI findings by an experienced radiologist, as well as similar sensitivities for both imaging modalities for discriminating between benign and malignant tumours (Shimada et al., 2018). The addition of diffusion-weighted techniques to conventional imaging modalities has been shown in multiple pooled studies to increase diagnostic accuracy in discriminating between benign tumours and ovarian cancer, especially in the Caucasian population, with data even suggesting a value in predicting resectability (Dai et al., 2019; Espada et al., 2013; Meng et al., 2016; Michielsen et al., 2017; Rizzo et al., 2020). However, the true extent of such a benefit needs to be validated further in multicentre, large-scale prospective randomized studies, which are currently being designed or underway (Michielsen et al., 2017). The addition of quantitative dynamic contrast-enhanced MRI to diffusion-weighted imaging and anatomical MRI sequences and the development of a 5-point scoring system (O-RADS MRI score) is another modern diagnostic development with promising potential for the differentiation between benign and malignant adnexal masses in cases in which ultrasound is unable to arrive at a clear diagnosis (i.e. indeterminate masses). When this technique is enhanced with volume quantification, it can help to discriminate between Type-I and Type-II epithelial ovarian cancers (Carter et al., 2013; Gity et al., 2019; He et al., 2020; Li et al., 2018; Malek et al., 2019; Thomassin-Naggara et al., 2012; Thomassin-Naggara et al., 2020). However, there are only limited data available on the impact of these modern MRI techniques on clinical decision- making and further studies are needed, with larger sample populations (Dirrichs et al., 2020).

#### Computed tomography

Dedicated multidetector CT protocols with standardized peritoneal carcinomatosis index forms are the most common diagnostic tool used in routine clinical practice to assess the extent of tumour dissemination and the presence of peritoneal carcinomatosis (Ahmed et al., 2019; Byrom et al., 2002; Esquivel et al., 2010; Marin et al., 2010; Nasser et al., 2016). A radiological peritoneal carcinomatosis index applied at preoperative CT within an expert setting has been shown to have low performance scores as a triage test to identify patients who are likely to have complete cytoreduction to no macroscopic residual disease (Avesani et al., 2020). On retrospective analysis, preoperative CT imaging showed high specificity but rather low sensitivity in detecting tumour involvement at key sites in ovarian cancer surgery (Nasser et al., 2016). Multiple studies that have attempted to cross-validate the accuracy of CT scans in predicting unresectable disease and incomplete cytoreduction have shown a substantial drop in accuracy rates when attempts have been made to validate them in other cohorts (Axtell et al., 2007; Bristow et al., 2000; Dowdy et al., 2004; Gemer et al., 2009; Kim et al., 2014; Nelson et al., 1993; Rutten et al., 2015; Shim et al., 2015). Thus, CT should not be used as the sole tool to predict the resectability of peritoneal carcinomatosis and exclude patients from surgery; rather, the full clinical context should be taken into account. Its widespread availability makes CT useful as a first- line diagnostic tool to identify patients who should not be selected for cytoreductive surgery, such as those with large/multifocal intraparenchymatous distant metastases, acute thromboembolic events or secondary metastatic tumours that limit the prognosis. The role of radiomics as an additional quantitative mathematical segmentation of conventional preoperative CT images has shown some promising results in preliminary studies; however, larger studies are necessary for validation before this technique is implemented in clinical practice (Lu et al., 2019).

#### Positron emission tomography-computed tomography

Positron emission tomography-computed tomography (PET-CT) may be useful in differentiating malignant from borderline or benign ovarian tumours, with the limitation that its diagnostic performance can be impacted negatively by certain tumour histological subtypes, due to the lower fluorodeoxyglucose uptake in clear-cell and mucinous invasive subtypes (Castellucci et al., 2007; Kitajima et al., 2011; Nam et al., 2010; Risum et al., 2007; Tanizaki et al., 2014; Yamamoto et al., 2008). PET-CT can also play a role as an additional technique in the diagnosis of lymph- node metastases, especially outside the abdominal cavity, or in characterizing unclear lesions in key areas that would alter clinical management, for example chest lesions (Dauwen et al., 2013; Kim & Lee, 2018; Laghi et al., 2017). However, PET-CT does not seem to be a relevant additional diagnostic modality for the true extent of peritoneal spread of ovarian cancer, specifically bowel and mesenteric serosa, and therefore fails to predict resectability in those key sites, especially in the presence of low-volume disease (Michielsen et al., 2014). Furthermore, PET-CT has been shown to have a low diagnostic value in differentiating borderline from benign tumours and should therefore not be used in clinical decision-making processes in that context, especially when considering fertility- sparing procedures (Kitajima et al., 2011; Tanizaki et al., 2014; Yamamoto et al., 2008).

**Statements on MRI, CT and PET-CT (Statements 13–17)**

-
  MRI with the inclusion of the functional sequences, dynamic contrast-enhanced and diffusion-weighted MRI, is not a first-line tool but may be used as a second-line tool after ultrasonography to further differentiate between benign, malignant and borderline masses.
  - Level of evidence: 2a
  - Grade of statement: B
  - Consensus: yes, 100% (n = 20); no, 0% (n = 0); abstain, 0% (n = 0)
-
  PET-CT and whole-body diffusion MRI as a second step can help to detect non-ovarian origin of secondary metastatic tumours if suspicions are raised by the initial ultrasound examination.
  - Level of evidence: 4
  - Grade of statement: C
  - Consensus: yes, 90% (n = 18); no, 0% (n = 0); abstain, 10% (n = 2)
-
  PET-CT cannot differentiate reliably between borderline and benign tumours.
  - Level of evidence: 4
  - Grade of statement: C
  - Consensus: yes, 95% (n = 19); no, 0% (n = 0); abstain, 5% (n = 1)
-
  Imaging alone cannot detect reliably the entire extent of either peritoneal carcinomatosis (especially in cases of small-volume carcinomatosis) or mesenteric and bowel serosal involvement.
  - Level of evidence: 3b
  - Grade of statement: B
  - Consensus: yes, 85% (n = 17); no, 5% (n = 1); abstain, 10% (n = 2)
-
  Imaging alone should not be used for surgical decision-making in terms of the prediction of peritoneal tumour resectability.
  - Level of evidence: 3b
  - Grade of statement: B
  - Consensus: yes, 80% (n = 16); no, 15% (n = 3); abstain, 5% (n = 1)

Levels of evidence and grades are described in Table III.

### Circulating cell-free DNA and circulating tumour cells

Circulating cell-free DNA and circulating tumour cells as non-invasive cancer biomarkers and in non-invasive biopsy (sometimes called ‘liquid biopsy’) have been investigated in multiple studies (Barbosa et al., 2018; Chen et al., 2019; Giannopoulou et al., 2018; Guo et al., 2018; Kolostova et al., 2015; B. Li et al., 2019; N. Li et al., 2019; Lou et al., 2018; Phallen et al., 2017; Suh et al., 2017; Vanderstichele et al., 2017; Widschwendter et al., 2017; Yu et al., 2019; Zhou et al., 2016). DNA methylation patterns in cell- free DNA show potential to detect a proportion of ovarian cancers up to 2 years in advance of diagnosis. They may potentially guide personalized treatment, even though validation studies are lacking. The prospective use of novel collection vials, which stabilize blood cells and reduce background DNA contamination in serum/plasma samples, will facilitate the clinical implementation of liquid biopsy analyses (Widschwendter et al., 2017). A prospective evaluation of the potential of cell-free DNA for the diagnosis of primary ovarian cancer using chromosomal instability as a read-out suggested that this might be a promising method to increase the specificity of the presurgical prediction of malignancy in patients with adnexal masses (Vanderstichele et al., 2017). However, even though these circulating biomarkers play a key role in understanding metastasis and tumorigenesis and provide comprehensive insight into tumour evolution and dynamics during treatment and disease progression, they still have not been established as part of routine clinical practice (Barbosa et al., 2018; Chen et al., 2019; Giannopoulou et al., 2018).

One meta-analysis suggested that quantitative analysis of cell-free DNA has unsatisfactory sensitivity but acceptable specificity for the diagnosis of ovarian cancer (Zhou et al., 2016). In a more recent meta-analysis, cell-free DNA appeared to be slightly better than CA 125 and similar to HE4 with respect to its diagnostic ability to discriminate individuals with from those without ovarian cancer (Li et al., 2019). Nevertheless, the diagnostic value of cell-free DNA in ovarian cancer patients remains unclear and the data should be interpreted with caution. Further large-scale prospective studies are strongly recommended to validate the potential applicability of using circulating cell-free DNA, alone or in combination with conventional markers, as a diagnostic biomarker for ovarian cancer, and to explore potential factors that may influence the accuracy of ovarian cancer diagnosis (Zhou et al., 2016).

**Statement on circulating cell-free DNA and tumour cells (Statement 18)**

-
  Circulating cell-free DNA and circulating tumour cells should not yet be used in routine clinical practice to differentiate between benign and malignant ovarian masses.
  - Level of evidence: 4
  - Grade of statement: C
  - Consensus: yes, 85% (n = 17); no, 5% (n = 1); abstain, 10% (n = 2)

Levels of evidence and grades are described in Table III.

## Overview of consensus

**Figure 2:**
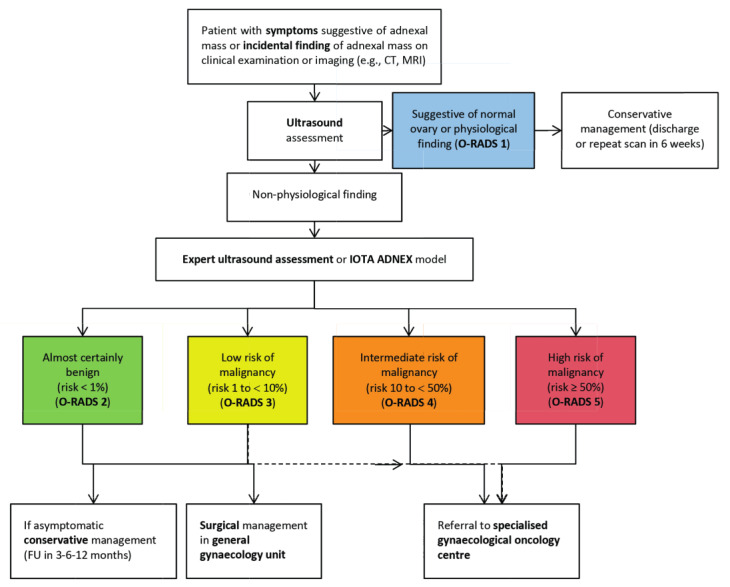
— Flowchart of steps recommended to distinguish between benign and malignant tumours and to direct patients towards appropriate treatment pathway. CT, computed tomography; F/U, follow-up; IOTA ADNEX, International Ovarian Tumour Analysis Group Assessment of Different NEoplasias in the adneXa; MRI, magnetic resonance imaging; O-RADS, Ovarian-Adnexal Reporting and Data System.

The experts also reached a consensus on a flowchart describing steps recommended to distinguish between benign and malignant tumours (Figure 2) and to direct patients towards appropriate treatment pathways. Ultrasonography is recommended as a first step to stratify patients with symptoms suggestive of an adnexal mass, and in those with an incidental finding of an adnexal mass on imaging. If the scan rules out normal ovaries and physiological changes (i.e. rules out O-RADS 1), the IOTA ADNEX model could be applied as a next step in order to determine the risk of malignancy. Any ultrasonographic examination in the case of a suspected ovarian mass should be performed by an expert sonographer. The resulting classification of the lesion into one of the O-RADS categories (2–5) can further guide the management and selection of patients for referral to a dedicated gynaecological oncology centre.

**Figure 3:**
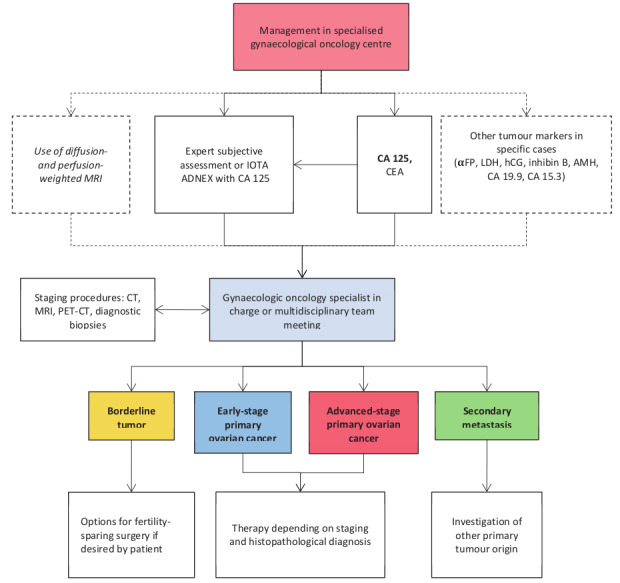
— Flowchart of steps necessary to differentiate between subgroups of malignancy and extent of disease within gynaecological oncology centres. *Early stage and advanced stage might differ according to different ADNEX models (Stage I vs Stages II–IV) and oncologically (Stages I–II vs Stages I–IV). αFP, alpha-fetoprotein; AMH, anti-Müllerian hormone; CA 125, cancer antigen 125; CA 15-3, cancer antigen 15-3; CA 19.9, carbohydrate antigen 19-9; CEA, carcinoembryonic antigen; CT, computed tomography; hCG, human chorionic gonadoptrophin; IOTA ADNEX, International Ovarian Tumour Analysis Group Assessment of Different Neoplasias in the adneXa; LDH, lactate dehydrogenase; MRI, magnetic resonance imaging; PET-CT, positron emission tomography-computed tomography.

A consensus was also reached on further steps necessary to differentiate between subgroups of malignancy and extent of disease within gynaecological oncology centres (Figure 3). Ultrasound assessment by an expert or application of the IOTA ADNEX model in combination with the tumour marker profile (CA 125 and CEA, complemented with other markers in specific cases) can often indicate the specific subtype of malignancy. If available, diagnosis of the primary lesion can be confirmed with diffusion- and perfusion-weighted MRI, especially in cases in which fertility-sparing surgery is considered. A CT scan of chest, abdomen and pelvis is mandatory before planned surgery for presumed malignancy, in order to exclude secondary cancers, thromboembolic events, and multifocal intraparenchymal distant metastases that would preclude operability. The final management and treatment journey of the patient should be determined within an expert multidisciplinary setting, taking into account both the diagnostic findings and the overall patient profile, including symptoms, patient preferences and prior surgical, medical and reproductive history, with the ultimate aim of defining an individualized approach for every patient.

## Appendix 1

**Identification of scientific evidence: literature search in MEDLINE.**

Research period
1 May 2015–1 May 2020
Indexing terms
adnexal masses, alpha fetoprotein, assessment of different neoplasias in the adnexa, assessment of different neoplasias in the adnexa masses, assessment of different neoplasias in the adnexa model, benign ovarian masses, benign ovarian tumours, beta-human chorionic gonadotropin, biomarker, borderline tumours, carbohydrate antigen 19.9, carbohydrate antigen 125, carcinoembryonic antigen, cell-free deoxyribonucleic acid, circulating cancer cells, circulating cell-free deoxyribonucleic acid, circulating free deoxyribonucleic acid, circulating tumour cells, circulating tumour deoxyribonucleic acid, clinical routine, computed tomography, consensus statement, daily practice, diagnosis, diagnostic performance, diagnostic models, diffusion-weighted imaging, diffusion-weighted magnetic resonance imaging, dynamic contrast-enhanced magnetic resonance imaging, expert ultrasound examiners, first line test, functional sequences, gynaecology imaging reporting and data system, human epididymis protein, imaging, imaging methods, immunohistochemical diagnosis, inhibin, international ovarian tumour analysis, international ovarian tumour analysis methods, international ovarian tumour analysis rules, intraoperative ultrasound, investigations, logistic regression 1 test, logistic regres- sion 2 test, magnetic resonance imaging, malignant ovarian masses, malignant ovarian tumours, marker, maximum standardized uptake value, molecular biology, molecular marker, morphological scoring system, multivariate analysis, ovarian cancer, ovarian masses, ovarian tumours, ovary, positron emission tomography, positron emission tomography-computed tomography, pre-operative characterization, pre-operative diagnosis, prognostic factor, prognostic value, protein biomarker, risk factors, risk of malignancy score, risk of malignancy index, risk of ovarian malignancy algorithm, scoring system, screening test, secondary metastatic tumours, second line test, simple rules, simple rules risk, simple rules risk model, single protein biomarker, standardized uptake value, suspected malignancy, suspected metastatic tumour, test performances, threshold risk, transabdominal ultrasound, transvaginal ultrasound, tumour markers, ultrasonography, ultrasound, ultrasound (3D), ultrasound-based diagnostic models, ultrasound-based risk models, ultrasound examiners, vascular endothelial growth factor, vascular endothelial growth factor, whole body diffusion magnetic resonance imaging.
Language
English
Study design
Priority was given to high-quality systematic reviews, meta-analyses and validating cohort studies, but lower levels of evidence were also evaluated. Search strategy excluded editorials, letters and case reports. Reference list of each identified article was reviewed for other potentially relevant papers.
